# In vivo imaging of therapy-induced anti-cancer immune responses in humans

**DOI:** 10.1007/s00018-012-1159-2

**Published:** 2012-10-05

**Authors:** Erik H. J. G. Aarntzen, Mangala Srinivas, Caius G. Radu, Cornelis J. A. Punt, Otto C. Boerman, Carl G. Figdor, Wim J. G. Oyen, I. Jolanda M. de Vries

**Affiliations:** 1 Department of Tumor Immunology, Nijmegen Centre for Molecular Life Sciences, Radboud University Nijmegen Medical Centre, PO Box 9101, 6500 HB Nijmegen, The Netherlands; 2 Department of Medical Oncology, Radboud University Nijmegen Medical Centre, Nijmegen, The Netherlands; 3 Department of Nuclear Medicine, Radboud University Nijmegen Medical Centre, Nijmegen, The Netherlands; 4 Department of Molecular and Medical Pharmacology, David Geffen School of Medicine, UCLA, Los Angeles USA; 5 Department of Medical Oncology, Academic Medical Centre, Amsterdam, The Netherlands

**Keywords:** Immunotherapy, Functional imaging, Dendritic cells, PET, Scintigraphy, MRI

## Abstract

Immunotherapy aims to re-engage and revitalize the immune system in the fight against cancer. Research over the past decades has shown that the relationship between the immune system and human cancer is complex, highly dynamic, and variable between individuals. Considering the complexity, enormous effort and costs involved in optimizing immunotherapeutic approaches, clinically applicable tools to monitor therapy-induced immune responses in vivo are most warranted. However, the development of such tools is complicated by the fact that a developing immune response encompasses several body compartments, e.g., peripheral tissues, lymph nodes, lymphatic and vascular systems, as well as the tumor site itself. Moreover, the cells that comprise the immune system are not static but constantly circulate through the vascular and lymphatic system. Molecular imaging is considered the favorite candidate to fulfill this task. The progress in imaging technologies and modalities has provided a versatile toolbox to address these issues. This review focuses on the detection of therapy-induced anticancer immune responses in vivo and provides a comprehensive overview of clinically available imaging techniques as well as perspectives on future developments. In the discussion, we will focus on issues that specifically relate to imaging of the immune system and we will discuss the strengths and limitations of the current clinical imaging techniques. The last section provides future directions that we envision to be crucial for further development.

## Introduction

Immunotherapy aims to re-engage and revitalize the immune system in the fight against cancer. A recent series of successes has indicated the broad potential of this approach and has led to the approval of several novel immunotherapies [89, 102, 125]. Although the recent progress is exciting, the underlying mechanisms are only partly understood [45].

Considering the enormous effort and costs involved in developing, optimizing, and applying an effective immunotherapeutic approach, it is remarkable that a monitoring tool that accurately identifies a responding patient early during immunotherapeutic treatment is lacking. Research over the past decades has shown that the relationship between the immune system and human cancer is complex, highly dynamic, and variable between individuals [107]. Given the diversity in immune responses among individual patients to a single immunotherapeutic intervention, every clinical case potentially provides a unique opportunity to understand the crucial processes that precede the failure or success of immune responses. In this respect, individualized medicine is not only a goal in itself but rather a tool to develop successful therapy. Therefore, further progress can be expected only if we manage to take this opportunity and learn how to guide therapy based on individual responses. The development of a clinically applicable tool to monitor therapy-induced immune responses in vivo is thus most warranted.

However, development of such a tool is complicated by the fact that a developing immune response encompasses several body compartments, e.g., peripheral tissues, lymph nodes (LN), lymphatic and vascular systems, as well as the tumor site itself. Moreover, the cells that comprise the immune system are not static but constantly circulate through the vascular and lymphatic system.

Current attempts to find such a monitoring tool often use surrogate markers, such as control antigens, or focus on a single functionality of immune effector cells, e.g., interferon gamma (IFNγ) enzyme-linked immunosorbent assays (ELIspots). In both cases, the results do not accurately link immune responses to clinical outcome. Furthermore, current immune-monitoring assays are based on peripheral blood cells or tissue and are therefore invasive. Novel techniques allow high-throughput assessment of individual variations in functional processes, e.g., differences in signaling pathways in immune cells [151]. As of now, these techniques lack validation and are not yet applicable to the evaluation of therapy-induced responses. In general, the assays currently available provide only snapshots of a continuous and dynamic process. Moreover, most assays attempt to either extrapolate the findings in individual subjects to the general treated population, or to interpret findings in individual patients based on previous findings in the general population. Thus, in order to obtain a more complete picture, we require new tools; the ideal monitoring tool should be non-invasive, allow longitudinal data acquisition, and reveal critical immunological processes that occur early during a treatment course on an individual basis. Quantification would be a further asset.

**Fig. 1:**
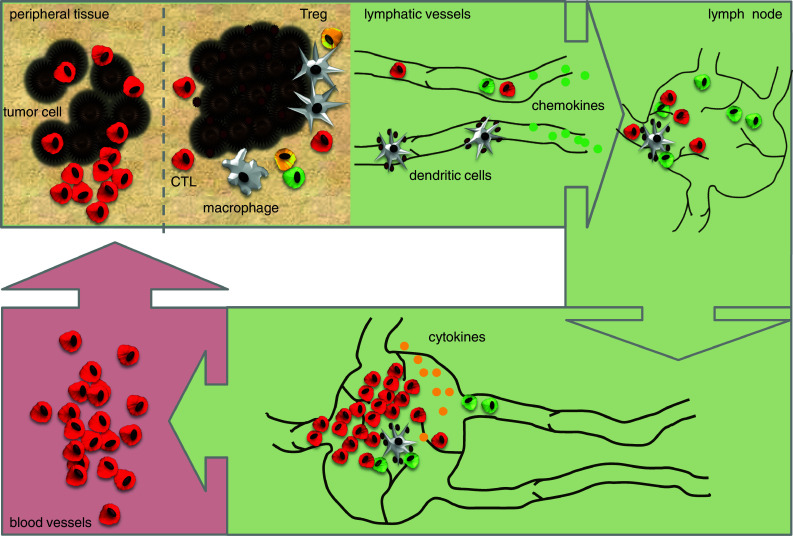
Overview strategies, response, and imaging

Molecular imaging is considered the favorite candidate to fulfill this task. The progress in imaging technologies and modalities has provided a versatile toolbox (Fig. 1) to address the issues mentioned above. Imaging modalities are available to image functional processes from a molecular scale to whole body levels.

This review focuses on the detection of therapy-induced anticancer immune responses in vivo and provides a comprehensive overview of clinically available imaging techniques as well as perspectives on future developments. We begin with an overview of the de novo immune response and the current therapeutic approaches that intervene at a given phase of the response. Next, we describe the possible target processes and the imaging techniques available within each phase of the developing immune response. In the discussion, we will focus on issues that specifically relate to imaging of the immune system and we will discuss the strengths and limitations of the current clinical imaging techniques. The last section provides future directions that we envision to be crucial for further development.

## Current immunotherapy approaches

The immune system is a highly organized multi-cellular system designed to protect the host from invading pathogens and malignantly transformed cells [13]. As such, the immune system acts with enormous specificity and great sensitivity, in concert with regulatory mechanisms, to avoid destructive self-reactivity. Many cell types from both innate (e.g., natural killer (NK) cells and macrophages) and adaptive (e.g., dendritic cells (DC), B cells and T cells) immunity contribute to immune competency. T cells are often considered to be the most critical effector cells in anti-cancer responses, since these cells are capable of developing antigen-specific memory responses. There are two major subsets of T cells, defined by the expression of the CD4 and CD8 surface markers. CD4^+^ T cells are subdivided to T helper 1 (Th1) cells, which activate macrophages, antigen-presenting cells, and cytotoxic CD8^+^ T cells to promote cellular immunity. T helper 2 (Th2) cells promote antibody production by activating B cells. Within the CD4^+^ T cell repertoire, a subset of T cells has the plasticity to become regulatory T cells (Treg), which mediate peripheral tolerance in physiological conditions. Although CD8^+^ cytotoxic T cells (CTLs), as the endpoint effector cells, represent a critical population for anticancer immunity, it has become clear that only a concerted action involving other cell types such as T helper cells and NK cells can result in an effective anticancer clinical response. In this respect, the secretion of small molecules like cytokines and chemokines is an important means of short- and long-distance communication between cells.

**Fig. 2:**
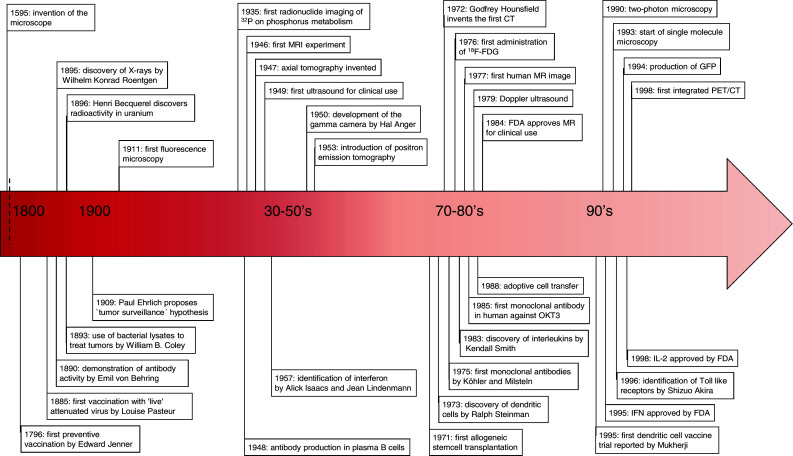
Timeline of development of immunotherapies and development of imaging tools

Developing tumors are often infiltrated by lymphocytes that specifically recognize tumor-associated antigens (TAA), but apparently are incapable of tumor eradication. However, these tumor-infiltrating lymphocytes can exert specific effector functions when disconnected from the suppressive tumor milieu. Based on this long-standing observation, the mainstay of immunotherapy is to induce, enhance, or sustain such tumor-specific cellular immune responses in order to overcome the suppressive environment at the tumor site. To achieve this, a plethora of strategies has been tested in preclinical models. Current immunotherapy approaches, which have been tested in clinical trials, intervene at different phases of a developing immune response. The next sections provide an overview of a developing anticancer immune response, divided in phases, and the current immunotherapeutic strategies that intervene at these phases, with a focus on HLA-restricted approaches (Fig. 2).

### Phase I: Antigen encounter

The development of human tumors is a multistep process that occurs over an extended length of time [59]. Since tumor cells originate from a normal cell and evade the immune system; human tumors express self-antigens that are poorly immunogenic, and lack pathogen-associated molecular patterns (PAMPs). As such, they rarely trigger robust inflammatory responses, especially when compared to the response to invading pathogens. Peripheral tissues are constantly screened by specialized antigen-presenting cells (APC) such as Langerhans cells and DC, which act as the sentinels of the immune system [13]. These immature APC are phagocytic and capture antigens. Small numbers of APC migrate to draining LN and present the processed TAA in major histocompatibility (MHC) complexes to effector cells. In the absence of inflammation, these APC remain in an immature state and ineffectively activate T cells. This absence of co-stimulatory signals and inflammatory cytokines leads to a tolerogenic T cell response. However, immature APC that encounter antigens under inflammatory conditions undergo a transformation to mature APC, which are highly migratory and potent orchestrators of adaptive immune responses.

#### Tumor antigen-containing vaccines

Following the identification of several TAA, a series of clinical trials has evaluated cancer vaccines that deliver TAA to neutral sites like skin or muscle, similar to preventive vaccines, in order to be recognized and phagocytosed by endogenous APC. These vaccines can consist of TAA peptides alone, in combination with immune stimulatory adjuvants like TLR-agonists and chemical agents (e.g., complete Freund’s adjuvant (CFA) or Montanide) [123], or complexed with immunogenic viral particles. A recent single-arm clinical study demonstrated that vaccination with long peptides derived from human papilloma virus (HPV)-16 E6 and E7 antigens induced complete tumor regressions in HPV-associated preneoplastic lesions [72]. In order to use the full repertoire of possible immunogenic epitopes, autologous or allogeneic tumor cells can be modified to provide immunostimulatory signals together with TAA [146].

#### Cellular vaccines

The isolation of specialized APC for ex vivo loading with antigen and activation provides a more controlled setting [12]. A recent phase III trial involving antigen-loaded antigen-presenting cells in patients with advanced prostate cancer demonstrated improved overall survival with vaccinations compared to placebo [68]. We have extensively studied the immunological responses to autologous antigen-loaded DC [48, 88]. Another highly interesting approach is the in vivo targeting of APC, which would replace laborious and expensive ex vivo culturing and facilitate large-scale application of DC-based vaccination therapies [142].

### Phase II: Expansion of immune effector cells

#### Cytokine-based immunotherapy

Soluble signaling molecules, e.g., cytokines, play an important role in the induction of inflammatory responses since they allow recruitment of lymphocytes and APC to the LN. Next, in the interaction of APC with lymphocytes in LN, cytokines provide a directive signal to the immune effector cells to skew their differentiation. Lastly, cytokines may have a direct antitumor effect or at least induce inflammatory responses at the site of the tumor.

The important role of cytokines prompted the administration of cytokines as anti-cancer immunotherapy. Interleukin-2 (IL-2), first described in 1976 as a T cell growth factor [143], plays a central role in immune regulation and T cell proliferation [130]. IL-2 was approved by the US Food and Drug Administration (FDA) in 1998 for treatment of advanced metastatic melanoma and in 2005 for the treatment of metastatic renal cell cancer. High-dose bolus intravenous IL-2 injections demonstrated antitumor effects [9, 109]. Long-term follow-up of these studies demonstrated durable responses in 4 % of the patients [2] suggesting the establishment of a memory T cell response. However, the side-effects associated with this treatment regimen are severe and often require hospitalization [30]. Interferon alfa (IFN-α) was the first exogenous cytokine to demonstrate antitumor activity in advanced melanoma. Interferon α-2b, a type I IFN, is a highly pleiotropic cytokine with immunoregulatory, as well as direct antitumor properties in multiple malignancies [54]. In 1995, interferon-α-2b became the first immunotherapy approved by the FDA for the adjuvant treatment of high-risk primary melanoma and objective responses were observed in approximately 15 % of patients with metastatic melanoma. However, due to its multiple effects, tolerability is an issue with this regimen and has hampered widespread use. Moreover, both IL-2 and IFNα have not shown a clear benefit in overall survival, neither in the adjuvant setting nor in metastatic disease.

#### Adoptive T cell transfer

Other than strategies to prime TAA-specific T cells in vivo, by cellular vaccines or cytokines, TAA-specific T cells can be isolated from tumor tissue or peripheral blood and primed in vitro to enhance their effector function. Adoptive cell transfer (ACT) involves the administration of tumor-specific T cells. The earliest form of ACT that demonstrated effective anti-tumor immunity was allogeneic bone marrow transplantation for chronic myeloid leukemia [93, 112]. Later studies have revealed that after T cells, alloreactive NK cells play a pivotal role in the graft versus leukemia effect [148]. Following the finding that T cells isolated from tumor or tumor-draining LNs can elicit a specific antitumor effect in vitro, this approach has been investigated in solid tumors. Transferred TAA-specific T cells are either isolated from patient tumor biopsies and expanded in vitro under immune stimulating conditions, or peripheral blood T cells are endowed with antigen-specific T cell receptors (TCRs) or fusion proteins termed chimeric activation receptors (CARs) [20, 119]. The expanded numbers of TAA-specific T cells in ACT should break immune tolerance at the tumor level and regenerate a broad tumor-specific immune response. Clinical trials show that critical issues for further improvement of this approach are the ex vivo re-programming of TILs towards a pro-inflammatory phenotype, sufficient T cell homing to the tumor and the avidity of T cells involved for in vivo expressed TAA [46, 66].

### Phase III: Targeting the tumor and its microenvironment

#### Monoclonal antibodies

Several immunotherapeutic strategies exploit direct antitumor effects, instead of indirectly by enhancing cellular immunity as mentioned above. The most prominent are monoclonal antibodies (mAbs) that can be divided into different classes according to their target (reviewed in [3]). First, mAbs are designed to activate the immune system by both antibody-dependent cellular cytotoxicity (ADCC), such as anti-CD20 mAb (rituximab) and complement-dependent cytotoxicity (CDC). Other mAbs inhibit cellular signaling pathways, such as the anti-Her2neu antibody trastuzumab, the anti-VEGF antibody bevacizumab and the anti-EGFR antibodies cetuximab and panitumumab. A novel promising antibody-based strategy is the use of bi-specific antibodies, which combine two different binding sites in order to bring specific effector cells in close proximity to specific target cells. For example, blinatumomab has a binding site for CD3, part of the TCR, and CD19, abundantly expressed on B cells. A recent phase II study demonstrated high response rates in patients with relapsed or refractory B-precursor acute lymphatic lymphoma [77].

#### Chemotherapy-induced immunogenic cell death

Evidence is accumulating that the immune system makes a crucial contribution to the antitumor effects of conventional chemotherapy-based and radiotherapy-based cancer treatments [8]. It has become clear that cell death induced by cytotoxic agents can be immunogenic and as such can trigger effective immune responses, reviewed in [163].

#### Overcoming tolerance

Strategies to neutralize immune suppressor mechanisms include chemotherapy (for example, low-dose cyclophosphamide), the use of antibodies (for example, CD25-targeted antibodies) in an attempt to deplete regulatory T cells and the use of antibodies against immune-checkpoint molecules (for example, cytotoxic T lymphocyte-associated protein 4 (CTLA4)-targeted antibodies and programmed cell death 1 (PD1)-targeted antibodies). Many of these strategies were recently reviewed [122, 132]. Ipilimumab, a mAb that blocks the inhibitory signaling by CTLA-4 that is expressed on activated T cells has demonstrated significant clinical efficacy in two recent large phase III trials [63, 118]. Based on these trials, ipilimumab has been approved by the FDA for metastatic melanoma [95, 125].

## General issues in clinical imaging

The recent development of novel immunotherapies warrants monitoring tools that allow accurate and early prediction of therapy response and disclose the critical preceding immunological mechanisms of action. In order to address these needs in clinical immunotherapy trials, the ideal monitoring tool is an imaging modality that allows whole-body, non-invasive, quantitative and longitudinal visualization of functional processes on a molecular level. However, in practice, a myriad of factors affect the choice of label and imaging modality for a specific application. Matching the right imaging system, including modality and probe, to an application is essential to its success. Multimodality imaging can maximize the strengths of each imaging modality while minimizing its weaknesses (Table 1), and is therefore being extensively explored. The next section describes the basic properties of clinically applicable imaging modalities.

**Table 1:** Characterization of clinically available imaging technologies for imaging immune responses

Modality	Spatial resolution	Temporal resolution	Sensitivity	Label lifetime	Functional information	Cell tracking	Anatomical information
MRI	50 μm–cm	s–min	Variable, generally medium to low	Variable	Yes	Ex vivo labeled cells	Yes
PET	mm–cm	min–h	Extremely high (picomolar)	h–days	Yes	In vivo	No
SPECT	mm–cm	min–h	Extremely high	h–days	Yes	Ex vivo	No
Planar scintigraphy	mm–cm	min–h	High	h–days	Yes	Ex vivo	No

### Scintigraphy

Planar scintigraphy is based on the detection of gamma radiation-emitting radionuclides, yielding 2D images. Most cell-tracking studies use scintigraphy because of the ability to quantifiable signal, lower cost, and wider availability. Scintigraphic imaging allows quantification of roughly greater than 10^4^ labeled cells, dependent on the amount of activity that can be loaded per cell [150]. However, scintigraphy lacks anatomic detail and is therefore increasingly being replaced by SPECT/CT. A key issue that arises when using radionuclides is the half-life of the label in relation to the lifespan of the transferred cells. The radiolabels that are typically used are ^111^In and ^99m^Tc, with half-lives of 2.8 days and 6 h, respectively, or ^18^F with a half-life of 2 h. This restricts the length of time that they can be detected in vivo (2–3 half-lives), often much shorter than the lifetime of the transferred cells. It also introduces logistic issues in planning such trials, as the entire process, from label synthesis to the final imaging, must be performed in a short period of time.

Furthermore, label retention within cellular compartments must also be characterized, as imaging modalities typically detect just the label regardless of whether the label is contained in the relevant cells, lost to the extracellular matrix, or transferred to other cells. For example, ^99m^Tc is not as suitable as ^111^In for labeling immune cells due to higher leakage of the ^99m^Tc label from the cells [22]. ^18^F-FDG has proven to be of little value in labeling transferred cells for in vivo tracking due to massive release from the cells [138], besides its short half-life.

### Magnetic resonance imaging

MRI uses powerful magnets to polarize and excite single protons predominantly in mobile water molecules, producing a detectable signal. It is a promising solution to the lack of anatomical detail in nuclear imaging modalities, since MRI provides excellent intrinsic contrast and high spatial resolution, even in soft tissues. MRI labels are comprised of very stable compounds such as superparamagnetic iron oxide (SPIO) or Gd agents. These heavy metals are chelated to reduce toxicity. Such labels are called “contrast agents” because they are not detected directly but instead through their effect on local contrast of mobile water in tissues. In this case, intracellular localization of label can also be important, particularly when using MRI labels where it has been shown that clustering of MRI labels in dense vacuoles yields better local contrast enhancement than cytosolic distribution [145]. Contrast agents have already been used in several clinical cell-tracking trials [31], allowing for high-resolution, longitudinal cell tracking. Finally, although MRI-based cell tracking is not restricted by radioactive decay as with radiolabels, this method is less suitable for quantification of cell numbers using current imaging protocols. Furthermore, the effect of these metals on the cells must be considered. Therefore, a novel class of ^19^F-based MRI contrast agents has been developed [136] with no physiological background and direct detection, which is suitable for quantification.

The development of novel imaging protocols and pulse sequences has led to the functional assessment of the tissue using techniques such as dynamic contrast-enhanced MRI (DCE-MRI) and diffusion-weighted imaging, which is discussed in later sections. The combination of anatomical information and functional information using a single imaging modality is very powerful and is therefore increasingly being investigated to monitor tumor responses to treatment.

### Positron emission tomography

PET reveals a three-dimensional image of functional processes as the system detects pairs of gamma rays emitted by a positron-emitting radionuclide. An advantage of PET tracers is that they are injected systemically, and then taken up by the relevant cells, as opposed to ex vivo labels as typically occurs with MRI, SPECT, and scintigraphy. Thus, the procedure can be carried out longitudinally (repeatedly), within the limits of radiation exposure. For example, patients were imaged with ^18^F-FLT PET at various time points to determine the peak of the DC-induced response [1]. However, in situ labeling requires high-sensitivity detection, typically through PET [81]. Another problem with systemic administration of tracer is the nonspecific accumulation in organs such as the kidneys or bladder, as well as uptake by irrelevant cell types, such as macrophages. ^18^F-FDG for example accumulates in the myocardium and in the brain and ^18^F-FLT accumulation is typically in the bone marrow.

It is important to consider the dosage and penetration of these injectable tracers, particularly given their short lifetime. Furthermore, tumors or other lesions can be susceptible to permeability changes from vascular disruption or leakage and this can affect the perceived signal intensity.

### Computed tomography

CT scans generate a three-dimensional image from a large series of two-dimensional X-ray images taken around a single axis of a subject. Its use has dramatically increased in the past decades due to increased availability and the circumvention of superimposition compared to planar X-ray images. The use of contrast agents has increased its diagnostic accuracy even further. For monitoring therapy-induced responses, CT scans provide accurate assessment of tumor volumes. X-ray beams are attenuated to different degrees by different tissue, resulting in image contrast. Based on the different attenuation of X-rays passing different tissues, a CT scan could provide information on the composition of tumors. Although changes in tumor composition are often observed on CT scans after initiation of treatment, this is not standardly used to evaluate treatment during clinical practice.

### Ultrasound

Ultrasonography uses high-frequency sound waves in the megahertz range that are reflected by tissue to varying degrees. While it may provide less anatomical detail than techniques such as CT or MRI, it has several advantages, in particular that it studies the function of moving structures in real time, emits no ionizing radiation, and is widely available and relatively cheap. On the other hand, its short penetration depth (in the centimeter range) and poor reproducibility are drawbacks for studying deep structures and its use in clinical trials. Doppler capabilities on modern scanners allow the blood flow in arteries and veins to be assessed, which can be further enhanced by the use of intravenous contrast agents, such as gas microbubbles.

## Immunological targets for imaging in humans

Given the enormous task of the immune system to maintain tolerance to self-antigens and yet induce immunity to potential harmful pathogens and malignantly transformed cells, it is obvious that immune responses are tightly regulated by intensive crosstalk between different immune cells. However, for the purpose of this overview, we have simplified this multistep process into a linear tri-phase sequel. The next section provides a description of immunological processes that have been used or potentially can be used as target for clinical imaging of developing immune responses. Each subsection highlights the specific contributions of the use of imaging to optimize therapy-induced immune responses.

### Phase I: Antigen encounter

The recognition and phagocytosis of antigens that are expressed by tumor cells represents the first step to induce an immune response (Fig. 2a). APC, e.g., DC, are specifically designed to fulfill this role. As such, APC are the main target for immunotherapeutic strategies exploiting TAA, either by in vivo loading or cellular therapy, using ex vivo generated antigen-loaded APC.

#### Labeling antigen-presenting cells in vitro

DC used in vaccination therapy are autologous cells, generally purified and differentiated from monocytes or from bone marrow. These DC are typically activated in vitro by adding pro-inflammatory cytokines or pathogen-associated danger signals, resulting in a phenotype with enhanced immune stimulatory properties [12, 48]. The therapeutic DC are loaded with tumor antigens before transfer back into the patient to induce antigen-specific responses. The ex vivo isolation of the DC allows convenient access for labeling before transfer. Most clinical studies have used ^99m^Tc or ^111^In to label the therapeutic cells for tracking in vivo, reviewed in [134].

Preclinical models have shown that the site of delivery greatly influences the subpopulation of DC that is targeted [47]. Moreover, the site of activation of lymphocytes dictates their preferential homing characteristics; skin-draining LNs induce skin-homing phenotypes in contrast to organ-draining LNs, which are involved in visceral homing lymphocytes [101, 103]. Lastly, the route of administration is important for the in vivo biodistribution of the transferred cells. The dermis is richly permeated with lymphatic vessels with loose endothelium accessible to DC. The subcutis consists of larger lymphatic vessels and has a different vasculature from the dermis, resulting in a less favorable milieu for DC to migrate. Imaging the therapeutic DC upon vaccination has revealed important clues on how to optimize vaccination protocols.

#### Optimizing the route of administration

Therapeutic DC have been administered by various routes in clinical trials: intralymphatic (i.l.), intravenously (i.v.), intradermally (i.d.), subcutaneously (s.c.), or intranodally (i.n.), and combinations of these. Intradermal vaccinations have been used commonly. Scintigraphic imaging of ^111^In-labeled DC shows that i.d. transfers result in a reproducible delivery of up to 4 % of the injected dose of mature DC to the LN, regardless of the activation conditions or mode of antigen loading. These small numbers of cells that reach the LN are sufficient to induce TAA-specific immune responses [149]. The results with s.c. administration are more variable, with only some studies detecting migration to LN, and always less than 4 %. Variations upon s.c. injections might be explained due to differences in injection techniques and the less favorable lymphatic structure of the subcutis. Intravenous administration results in a constant pattern of distribution in clinical studies, starting with entrapment in the capillaries of the lungs, which is most likely caused by non-specific activation and subsequent transient stiffening of cell membrane due to ex vivo handling of the cells. This is supported by the finding that mature DC are trapped in the lungs for a longer period of time than immature DC [113]. Thereafter, the DC redistribute mainly to the liver, spleen, and bone marrow. No LN localization has been detected, as expected, based on studies using normal peripheral blood leukocytes [116]. It is not clear whether DC actually completely fail to reach the LNs or whether the techniques used are not sufficiently sensitive to detect the small numbers of cells that do reach the LNs. Hence, DC migration to LN upon i.v. transfer has not yet been demonstrated in humans. These studies demonstrate that i.d. injections result in a reproducible delivery of a small, but potent, number of DC to the LN. It should be noted that localized transfers, such as i.d. or i.n. injections, are much easier to image than systemic transfers, due to the higher local cell densities, at least at the injection site.

#### Confirming accurate delivery of the vaccine

Intranodal administration is the most common in clinical studies, after i.d. injections. As the LN is the site of immune activation, delivery of DC directly into the LN obviates the need for specific and optimized migratory capacities and directly thrusts the entire dose of cells to the optimal location. The percentage of cells that migrate from the primary injected node to secondary nodes ranges from 0 to 84 %. This large variability cast doubts on the accuracy of i.n. injections, even when administered under ultrasound guidance. Therefore, we studied the migration and localization of a DC population dual-labeled with ^111^In and iron oxide using scintigraphy and high-resolution anatomic MRI [39]. Surprisingly, in four out of eight vaccinations we observed no migration to secondary LN, due to extranodal injection of the DC (confirmed using high-resolution MRI).

#### Labeling antigen-presenting cells in vivo

More challenging is in vivo labeling of antigen-presenting cells. Tagging DC in vivo through the use of labeled antigen is a convenient trick [17, 24]. For example, a nasal vaccine consisting of ^18^F-labeled botulinum neurotoxin was imaged in real time and in a quantitative manner using PET in primates [159]. In this study, the investigators demonstrated that nasal administration is safe with respect to spreading antigens to the central nervous system. Furthermore, whole-body PET allowed detection of the degradation of the vaccine over a period of 4 h. Long and colleagues exploited in situ labeling through cell-to-cell transfer. In a mouse model, they injected irradiated tumor cells labeled with superparamagnetic iron oxide (SPIO) and tracked the subsequent migration of APCs, which had taken up the label to draining LNs [92]. The cells could even be magnetically recovered ex vivo, allowing investigators to study the phenotype and functionality of the transplanted cells after injection. In humans, this can be done using ^111^In-labeled tumor antigen peptides and scintigraphy [82].

In another approach, the label is injected systemically and either taken up non-specifically by the relevant (phagocytic) cells or specifically by the relevant cell type. This approach circumvents the ex vivo purification and labeling of cells and is therefore much easier to apply and more amenable to use in large-scale studies. A simple example of such a label would be the use of a radiolabeled specific antibody bound to a radioactive isotope for SPECT [73]. Preclinically, this can be done using several techniques including MRI [5] and multimodal nanoparticles [21, 134]. However, in clinical practice, it has been proven difficult to achieve a sufficient signal in the relevant cells, which has so far hampered its use in human studies.

### Phase II: Expansion of immune effector cells

The LN are the key organ site in the interplay with DC for initiation of the ensuing immune response and remodeling of the LN infrastructure is an early event of this process [61, 62]. Murine studies showed that this occurs via endothelial cell (EC) activation and proliferation (Fig. 2b). VEGF is known to induce EC activation and is expressed on EC, DC, B cells, and T cells, in response to inflammatory cytokines [6, 11, 23, 100, 120, 160]. Expansion of the LN infrastructure, e.g., lymphangiogenesis and angiogenesis, facilitates the recruitment of immune cells and their proliferation. Enhanced influx and entry of DC enhances the immune response by ensuring ample antigen presentation. Next, increased influx and screening by naive T cells for specific antigens results in more potent responses [7, 131, 152]. Thus lymphangiogenesis, APC—lymphocyte interaction, and lymphocyte proliferation can serve as markers for immune responsiveness.

### Remodeling of the LN vasculature

It has been shown that LN volume can change nearly fivefold with induction of an immune response [76]. Direct imaging for LN volume is relatively straightforward, and can be done using various techniques visualizing the anatomy including MRI, CT, and ultrasound [15]. Recent data have shown that ultrasound imaging using targeted microbubbles improves the evaluation of the microvasculature, even in three dimensions [141]. The availability of gadolinium (Gd) or (ultrasmall) SPIO-based contrast agents allows the use of dynamic contrast-enhanced (DCE)-MRI to monitor angiogenesis on a functional level [79, 80, 85, 86]. DCE-MRI allows assessment of properties of LN vasculature such as expansion of LN size, total blood flow and blood volume, permeability of perfused capillaries, and total surface of perfused capillaries. To date, the main application of diffusion-weighted (DW)-MRI in imaging LNs is to detect metastatic LNs, for which it has proven high sensitivity. Accordingly, imaging reactive LNs in immune responses is logically the next application [76]. It has recently been shown that MRI measures of vascularity using an injected iron-based contrast agent are comparable to those obtained from traditional histology, which has long been the gold standard to study angiogenesis [87], thus validating the technique.

#### Indirect imaging of LN vasculature

Alternatively, lymphangiogenesis can be measured by targeted imaging of molecular markers [78]. The dominant events in the remodeling of newly formed blood vessels and lymph vessels, reviewed in [4], are coordinated by the expression of VEGF, and sprouting vessels abundantly express the α_v_β_3_ integrin. PET tracers have been developed to probe these specific targets. At our institute, the anti-VEGF antibody bevacizumab, labeled with ^111^In, is used for the scintigraphic detection of VEGF in tumors [139], but could easily be applied to image LN revascularization. More recently, a new generation of protein-targeted contrast agents for multimodal imaging of the cell-surface receptor for VEGF was described [10]. These probes are based on recombinant VEGF with a cysteine-containing tag that allows site-specific labeling with contrast agents for near-infrared fluorescence imaging, single-photon emission computed tomography (SPECT) or PET, reviewed in [33]. It is expected that an integrin targeting probe for PET will be available for clinical use in the next few years. Importantly, integrin-targeted PET probes have already been tested for safety in humans [74, 98]. α_v_β_3_ integrin has also been targeted by radiolabeled RGD-peptides, which specifically bind the integrin, an example of which is ^18^F-FPPRGD2 [98], ^18^F-galactoRGD [18] or ^18^F-Fluciclatide [16]. In mice, similar PET probes have been shown to be sensitive to antiangiogenic therapy [16]. Using a ^124^I-labeled antibody against the lymphatic vessel endothelial hyaluronan receptor-1 (LYVE-1), Mumprecht et al. [104] imaged inflammation-induced expansion and regression of lymphatic networks in vivo in mice with PET. MRI is also being explored in preclinical models to specifically target molecular markers such as α_v_β_3_ integrin [153, 156]. In clinical practice, enlargement of regional LN in response to preventive or therapeutic vaccination is a well-known phenomenon. Surprisingly, it has not yet been systemically investigated as a marker of immune responsiveness by using imaging modalities.

### Imaging lymphocyte activation in LNs

In order to stimulate lymphocyte proliferation, antigen-bearing mature DC must come into direct contact with the lymphocytes. This cell–cell interaction is best imaged using microscopic techniques, including intravital microscopy, which allow direct viewing. Imaging such specific cell–cell interactions in vivo has not yet been performed in clinical studies. However, preclinical studies have revealed important information on the dynamics and kinetics of critical interactions and have paved the way for clinical applications.

#### Imaging chemotaxis

Aimed at facilitating influx of both APC and effector cells, reactive LN express and secrete chemokines in order for immune cells to relocate to the reactive LN [7, 99]. Among others [114, 158], the presentation of chemokine CCR7 is dominant [14, 26] and provides a rational target for imaging. Chemotactic agents, which play a key role in directing trafficking, are also suitable imaging targets. CXCL12 is a key chemotaxis factor for lymphocytes, and is detected by CXCR4 on their cell membrane. CXCR4 overexpression is thought to play a role in cancer [83]. Thus, it has been explored as a potential imaging target. Imaging of surface receptors allows for in situ labeling of the lymphocytes, given that detection is highly specific and sensitive. Hence, PET is the most suitable approach, together with well-designed radioactive probes [40]. CXCR4 expression has been assayed in vivo in a dynamic manner using tagged ligands [55, 155], showing that CXCR4 can reliably and specifically be targeted in a manner that correlates with cellular composition shown by immunohistochemistry.

#### APC–lymphocyte interaction

The in situ dynamics of DC–T cell interactions have been studied extensively using advanced microscopy methods, which make it possible to study the kinetics of DC–T cell interactions (for example, [71]). Techniques such as intravital microscopy have enabled the study of immune cells in their native environment, with minimal external interference [25, 35, 50, 67, 75]. Single-molecule techniques now facilitate direct study of the relevant receptors and cell components at the T cell synapse [44]. It is now even possible to measure the forces generated by these molecular interactions ex vivo [26]. From these studies, it became clear that DC–T cell interactions are highly complex and precisely regulated events that govern immune responses. Such findings contribute to the concept that immune activation occurs in separate stages (static, dynamic), emphasizing the importance of motility (e.g., CCR7 expression) and chemokine secretion by LN stroma/cells and DC. With respect to DC-based immune therapy, these studies show that the life-span of DC, prolonged antigen presentation, and the migratory capacity are crucial for efficient immune induction. These extremely high resolution techniques are obviously restricted to ex vivo use.

#### Lymphocyte proliferation

Efficient stimulation by APC should result in lymphocyte activation and the subsequent release of cytokines, such as IL-2, together with extensive proliferation, is an energy-consuming process. Activated, antigen-specific lymphocytes then emigrate from the LNs to antigen depots. In terms of detection of the immune response, these changes in cell metabolism are a candidate for imaging. In particular, PET has been employed to study immune activation in vivo, as it allows the use of radiolabeled, injectable analogues of relevant metabolites, particularly glucose and nucleotides. Increased glucose uptake can be measured using ^18^F-labeled fluoro-2-deoxy-2-d-glucose (^18^F-FDG) PET, which is by far the most commonly used PET tracer. In hematolymphoid tissues, however, increased levels of deoxycytidine (DCK) expression is found; DCK is the rate-limiting step in the deoxycytidine salvage pathway. The tissue-specific expression of this enzyme allows more specific targeting by appropriate PET tracers [27]. For example, ^18^F–2-fluoro-d-(arabinofuranosyl)cytosine (^18^F-FAC), a fluorinated deoxycytidine analog, has been shown in animal models to accumulate preferentially in CD8^+^ T cells in mice studies. In contrast to ^18^F-FDG, this preferentially accumulated in innate immune cells [105].

The accumulation of nucleotide analogues, required for increased DNA synthesis during cell division, is another sensitive marker for antigen-specific lymphocytes, at least in melanoma patients vaccinated with antigen-loaded DC. ^18^F-labeled 3′-fluoro-3′-deoxythymidine (^18^F-FLT) is trapped intracellularly after phosphorylation by thymidine kinase 1 (TK-1). ^18^F-FLT-phosphate is not incorporated into DNA since ^18^F-FLT-monophosphate is a very poor substrate for the second kinase, thymidylate kinase (TMPK), and thus hampers procession to ^18^F-FLT-triphosphate, which can be incorporated into the DNA. The accumulation of nucleotide analogues has also been studied using other radiolabels in humans. In one study, an ^11^C-tagged thymidine analogue used for PET was compared to ^18^F-FDG-PET in lung cancer [96]. This study confirmed our results that nucleotide analogue ^18^F-FLT is more specific for detecting proliferation than ^18^F-FDG.

**Fig. 3:**
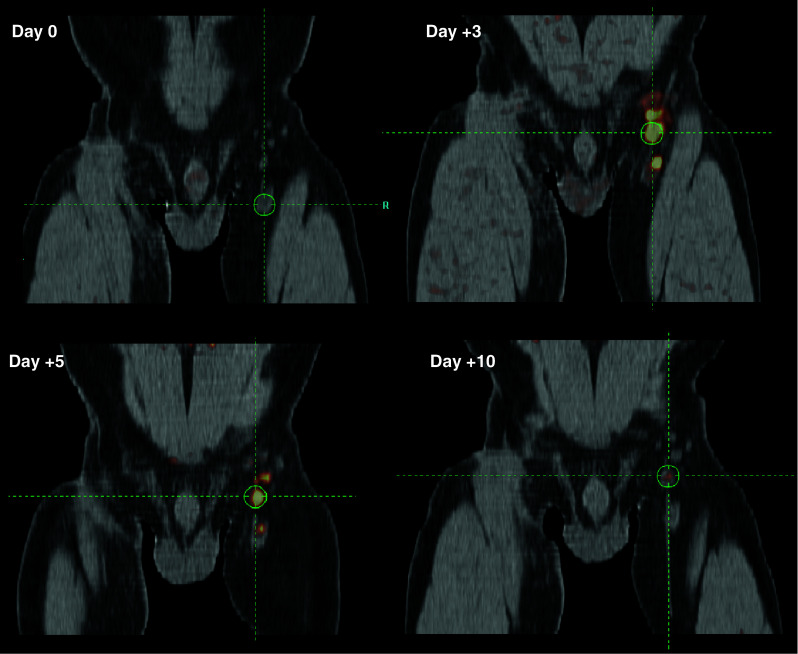
Example PET/CT scan of the dynamics of ^18^F-FLT uptake in LN after vaccination

In our study, we directly compared the properties of ^18^F-FDG and ^18^F-FLT within individual patients and demonstrated that in terms of sensitivity and specificity, both tracers perform similarly [1]. However, for ^18^F-FDG there was no correlation with the in vitro monitoring assays measuring concurrent antigen-specific T and B cell responses. Furthermore, ^18^F-FDG uptake can be attributed to other factors, including other treatments such as vaccinations [32]. In contrast, ^18^F-FLT retention in the LNs of vaccinated patients only increased in the presence of antigen-loaded DC. Moreover, the degree of increase directly correlated to the magnitude of the induced antigen-specific T and B cell responses. This was the first clinical demonstration in that antigen-specific therapy-induced immune responses can be imaged in vivo early after treatment initiation (Fig. 3. Example time course ^18^F-FLT uptake in vaccinated LN).

#### Imaging trafficking of immune effector cells

Activated lymphocytes must leave the LNs and migrate to sites where their cognate antigen is present. Imaging of lymphocyte trafficking is most easily achieved with ex vivo labeled cells, as in cases of ACT, but some clinical studies have explored in vivo labeling of effector cells as well.

#### Ex vivo labeling of transferred cells

Imaging of cells after ACT has recently been reviewed [115]. Transfused cells often traffic initially to the lungs, bone marrow, liver, and spleen [73], a process that is regulated by small molecules, primarily cytokines and chemokines. It has been demonstrated in early clinical trials that pretreatment with cyclophosphamide augments the trafficking of transferred cells to the tumor sites [111]. In a similar way, IL-2 co-administration might positively contribute to the vaccination effect [60].

The use of reporter gene expression as another way to study small-molecule expression is particularly exciting. It detects the actual synthesis of the molecule and is independent of factors such as lifetime and distribution of the molecule itself. Furthermore, the use of an enzymatic reporter allows for amplification of a weak signal. Hence, mice have been transfected with luciferase linked to interferon-β expression via plasmids for bioluminescence imaging [162]. In this study, the plasmids were injected directly in the liver. Such technology can be adapted to other molecules of interest. For example, antigen-specific T cells expressing a viral TK gene were tracked in recipient mice over a period of 3 weeks using an ^18^F-tagged probe specific to this variant of TK [127]. Quantitative detection of the labeled T cells was possible, with a sensitivity limit in the order of 10^4^ T cells—low enough to detect the milder immune response triggered by non-mutated self-antigens in cancer. However, nonspecific probe accumulation in the tumor complicated image interpretation.

#### In vivo imaging of effector cells

Imaging of lymphocytes, particularly T cells, has been carried out in vivo in preclinical models using several imaging modalities [56]. Lymphocyte imaging requires a suitable target for the imaging probe, such as cell surface markers. For example, ^99m^Tc-labeled IL-2 can be used to detect lymphocyte-associated lesions in melanoma patients [128]. This probe detects tumor-infiltrating lymphocytes, and such imaging can be used to study the effect of therapy. The technique can also be modified to target other immune cells, provided for example, with non-depleting ^111^In-labeled anti-CD4 antibodies to track CD4^+^ T cells, as in a murine model of colitis [69]. The signal measured by SPECT in this model was found to correlate with standard pathologic measures, although unlike standard pathology, the SPECT/CT measurements are noninvasive and can be applied in humans. A recent example is the use of in vivo ^19^F MRI to longitudinally and quantitatively track T cell homing to draining LN [137]. Here, the numbers of antigen-specific T cells in a relevant LN were quantified over a period of 3 weeks, in the same animal. Quantification errors arising from dilution of label due to cell division are unavoidable in such systems [135].

A unique situation arises for in vivo clinical imaging of the immune system in humans in the cornea, where advanced microscopy techniques such as confocal and dual-photon microscopy have been carried out on endogenous DC and lymphocyte infiltration [94]. The transparency and favorable optical properties of the eye makes this possible. However, the functionality of the immune system in the eye may not be directly comparable to that in other regions—it was long thought that the eye was “immune privileged” and lacked an immune system. In vivo imaging in the eye is more advanced in preclinical settings, which allows more manipulation and the use of pre-labeled cells [133].

### Phase III: Targeting the tumor and its microenvironment

Traditionally, measuring the change in tumor volumes according to the response evaluation criteria for solid tumors (RECIST) has been the key criterion upon the therapy effect is judged [144]. The use of volume as a measure for response to treatment is based on a large body of evidence involving chemotherapy. Indeed, the direct cytotoxic mode of action of chemotherapy often translates into tumor shrinkage weeks to months after the start of treatment. This initial volume response is often correlated to clinical outcome and thus justifies its use in clinical decision-making. However, now that immunotherapeutic strategies have entered clinical practice, it has become clear that the traditional RECIST criteria are challenged by the advance of immunotherapy [65].

In general, several factors need to be considered for the development and optimization of a clinical imaging protocol. For example, there are often no solid data to plan the optimal label or contrast dosage, timing, and frequency of imaging and interaction with drugs or other interventions. Frequently, there are not enough subjects to obtain results with statistical significance, especially given the high variability that can occur between patients. Thus, trials that incorporate novel imaging for response evaluation must be scrupulously planned beforehand, but can also yield valuable information on the mechanism of action of the applied therapy and on the early identification of responding subjects. The next sections describe several imaging strategies to dissect the relative contributions of tumor progression and on-site immune action.

#### Evaluation of tumor volume

Tumor shrinkage results from a complex interplay of various components of the immune system in different body compartments. In general, this takes weeks to months to develop and in this time frame the tumor will continue its expansive growth, giving misleading results when tumor size alone is measured. Secondly, in order to eliminate tumor cells, immune cells need to penetrate the tumor and its microenvironment to achieve cell–cell contact. This implies increased cellularity of the tumor and is, in terms of tumor volume, indicative for tumor progression. Wolchok et al. [154] proposed a new response paradigm that addresses these issues, while using volume-based criteria. They evaluated a novel set of response criteria in a large series of patients with advanced melanoma who received ipilimumab, a fully human monoclonal antibody that blocks CTLA-4 [154]. These immune-related response criteria now more accurately characterize new response patterns, especially those with delayed tumor shrinkage or initial tumor growth followed by tumor shrinkage. However, these novel criteria are designed to avoid preliminary termination of a possible effective immunotherapeutic treatment, but they fail to reveal cellular and molecular processes that precede a clinically meaningful response. Furthermore, as a result of the delayed volume responses that are associated with the indirect mode of action of immunotherapy, this information becomes available rather late after initiation of treatment.

#### Imaging tumor cellular composition

One rational approach to circumvent the above-mentioned issues is to measure the relative number of tumor cells in a suspected tumor-containing volume before and after the start of treatment. Such an approach requires a highly sensitive and quantifiable tumor-specific marker, e.g., by using PET tracers. The developments in melanoma-specific markers is recently reviewed in [97]. In this respect, novel compounds that target melanin biosynthesis and metallopeptides binding to melanocortin type 1 receptor, which are overexpressed in melanoma, are promising agents [41, 42, 57]. Changes that occur in the tumor due to an increased immune response can be imaged using MRI, for example through changes in relaxation times, contrast, or apparent diffusion coefficient. These changes have been shown to correlate with conventional histological measures in mice [84]. In this study, the immune response was induced by transferred cytotoxic T cells that expressed a modified TCR specific for a tumor antigen. However, to date, there is no experience with the evaluation of responses to immunotherapy in particular.

#### Imaging tumor metabolic activity

Currently, ^18^F-FDG is the most commonly used radiopharmaceutical for imaging tumor metabolism in clinical practice. Its use is based on the increased glycolytic rate in tumors compared to physiologic cells, known as the Warburg effect. Without doubt, imaging changes occurring in tumor metabolism early after treatment initiation by PET has contributed to the optimization of clinical decision-making in the management of patients with various types of cancer. However, the infiltration of effector immune cells, which are metabolically active as well, can be a confounder in the interpretation of tumor responses, leading to ^18^F-FDG-positive tumor lesions due to activated immune cells rather than tumor cells [140]. Regardless, it has also been shown through immunohistochemistry that highly ^18^F-FDG avid lesions that were not regressing indeed showed a high proliferative rate of tumor cells, whereas low ^18^F-FDG avid lesions were massively infiltrated by activated immune cells. There is increasing attention for the development of tracers which are more tumor-specific, in order to discriminate tumor metabolism from inflammatory responses. Potential candidates are amino acids, nucleotides, choline, and σ-receptor ligands. In a preclinical model, Van Waarde et al. [147] compared two σ-receptor ligands, ^11^C-methionine and ^11^C-choline, with ^18^F-FDG and found that one of the ^18^F-labeled σ-receptor ligands selectively targeted glioma metabolism; 30-fold tracer uptake compared to sterile inflammation. Further in vitro studies in this model showed that increased sigma-ligand binding and ^11^C-choline uptake reflected active membrane repair upon chemotherapy-induced cell damage.

Along the same lines, imaging nucleotide metabolism by ^18^F-FLT, as a tracer for tumor cell proliferation in humans, has extensively been evaluated to show proliferation specifically. Effector immune cells that infiltrate tumors are mostly of a differentiated phenotype and show no proliferative activity on the spot. However, no comparative studies in humans have been performed to study the relative selectivity of ^18^F-FLT for tumor cells compared to inflammation [28].

## Discussion and future directions

Direct visualization is a powerful tool to push forward the understanding of complex processes, which has intrigued researchers for ages (Fig. 1 Timeline). In the next section, we describe particular issues concerning the application of imaging immune responses in clinical practice. Understanding those issues facing the imaging therapy-induced anticancer immune responses will hopefully improve the usage of these tools in future clinical trials and thus contribute to the optimization of anti-cancer treatment.

### General issues in imaging the immune system

Germain et al. [50] eloquently stated that the imaging of the immune system is the biological equivalent of Heisenberg’s principle, which implies that it is not possible to study the system without perturbing it. Indeed, the function of the immune system is to maintain cellular integrity and homeostasis; hence, how can you label and probe it without affecting the system? Therefore, extra caution must be taken in the development of probes and labels for imaging. The highly mobile and rapidly changing nature of the immune system further complicates imaging studies. We have previously suggested two basic strategies for in vivo cell tracking [134]. Briefly, cells can be pre-labeled before transfer or a targeted label can be used to label the relevant cells in situ. Typically, the first strategy is used for MRI (pre-labeling with contrast agents), and the second for PET. This is a reflection of both the detectable lifetime of the labels and the sensitivity of the detection technique.

#### Ex vivo labeling of cells

From an imaging perspective, there are several advantages to this approach. First, the ex vivo isolation of the DC allows convenient access for labeling before transfer, and for detailed characterization of the labeled cells in terms of viability, gene expression, and functional status [119, 134]. Secondly, the homogeneity of label uptake within the population can also be determined and removal of excess label and dead cells is simpler. Furthermore, non-specific labeling of irrelevant cells is greatly reduced if the relevant cell population is purified beforehand. However, it can be an expensive and laborious process to purify, culture, and label cells ex vivo before transfer. The effect of an imaging label on cells must be carefully considered. For example, radioactive probes can become highly concentrated locally and affect the labeled cells directly [52]. This can be particularly deleterious for long-lived or highly proliferating cells, such as stem cells or activated T cells. MRI labels, such as those based on iron oxide, have also been shown to impact cell migration [36] [38] and induce oxidative stress due to the catalytic iron moiety [106]. The fate of the label, particularly its ability to stay with the relevant cell is crucial. It is known that cells can transfer their intracellular label to neighboring cells, particularly when under stress, leading to “secondarily labeled cells” that can confound imaging data [129]. Hence, one always should ask—and answer—the question “What am I imaging?” These cellular effects must be considered in addition to any systemic side-effects of the label, for example nausea or rashes; nephrogenic systemic fibrosis is a concern with Gd-based contrast agents for MRI.

#### In vivo labeling of cells

The alternatives to ex vivo labeling, using long-lived agents, are in situ labeling or genetic modification of the cells to express an imaging reporter gene. In situ labeling typically uses short-lived labels, as with injectable PET tracers. These tracers can even be generated from clinical applicable antibodies, for example ^89^Zr-labeled Fresolimumab for PET detection of tumor necrosis factor (TNF)-β expression in mice [108]. However, antibodies can neutralize or otherwise affect activity of the target molecule and/or cell. While that is often the purpose in antibody therapy, it is not necessarily desirable when imaging functionality or expression. In general, only viable or functional cells will be able to take up the label effectively, so non-specific labeling will not restrict its use. Furthermore, the use of an injectable label allows the use of radiolabels for longitudinal studies, as the agent can be injected (or re-injected) before each imaging session. However, the general problems faced with systemic transfer of label include limited uptake in the relevant cell population, accumulation in non-relevant tissues such as the liver or bladder, the requirement for higher activity doses to allow sufficient signal in the relevant cells, clearance of label and clinical radiation exposure limits for radioactive agents.

The use of reporter genes is a powerful approach. This technique is well suited for the detection of proliferative cells, as the label does not dilute with cell division. Furthermore, continued expression over the cell’s lifetime allows longitudinal tracking, and can even be coupled to the expression of a particular gene of interest, or at the very least, to viable cells. Moreover, intrinsically labeled cells are attractive due to the absence of background from nonspecific uptake [24]. However, the applicability of genetically modified cells to humans is not yet clear [34]. Finally, it is always necessary to consider whether images are really specific to the relevant cells, i.e., the specificity of detection. While this is typically more of a problem with injected labels or targeted agents that are injected systemically, it can also affect prelabeled cells through nonspecific transfer or loss of label from the transferred cells. The actual physical location of the label should be confirmed using histology on tissue sections during the development and validation of such monitoring tools.

### Future directions: how can imaging contribute?

The greatest challenge is to find a target process that is critical to successful immune activation, so that imaging this process accurately predicts response to intervention. Crucially, this must be done without disturbing the targeted process. Moreover, the imaging results should deliver important information as early after start of treatment as possible, to allow timely adjustment of treatment per individual. In this section, we describe three possible solutions, using novel imaging tools fulfilling the needs described above.

#### Imaging specific functionalities

PET reporter gene (PRG)/probe (PRP) systems, recently reviewed in [29, 157], have proven their use in many preclinical models [127]. PRG encodes a protein that mediates the accumulation of a specific reporter probe, labeled with positron-emitting radionuclide. These systems allow long-term whole-body visualization of the functional status of the transduced and transplanted cells, and can thus provide valuable information on the localization, kinetics, and magnitude of transgene expression over time. The application in patients has long been hampered by safety concerns; the most commonly used transgenes are from viral origin and can trigger an immune attack against the transfected cells in humans [19]. However, strategies to circumvent the safety concerns are being developed, paving the way to application in clinical trials. For example, therapeutic cells have genetically been modified to implement a suicide gene in ACT as a safety precaution. Another possibility to circumvent this immunogenicity is customizing the reporter gene, as has been done with thymidine kinase (TK) [34]. TK gene therapy has been applied in small clinical trials [121, 124] and the cells used in ACT are frequently genetically modified (for example [37, 43]). Recently, humanized transgenes were described [34, 90, 91], based on a mutated form of the human thymidine kinase 2 (TK2). By using this PRG and a thymidine analog L-^18^F-FMAU as PRP, the investigators could efficiently target and visualize nucleotide metabolism in proliferating cells without perturbing the endogenous enzymes. In this study, the biodistribution of the used PRP and another probe, ^18^F-FHBG, were studied in patients, as a first step towards clinical application. This promising technique can easily be adjusted to target other functional processes, from the monitoring of cell-based therapies to anti-angiogenic treatment. In animal models, the relevant cells can be transduced to express reporter genes, such as enzymes that trap tracers for PET, fluorescent proteins such as green fluorescence protein (GFP) for fluorescence imaging, luciferase for bioluminescence imaging, or iron transporters or CEST proteins [53] for MRI contrast, and even for multimodality imaging using a triple reporter gene construct for fluorescence (eGFP), ^18^F-FLT PET (TK 1 and 2) and luminescence (luciferase) [110].

#### Novel tracers that target specific effector cell populations

The activation and proliferation of immune effector cells is accompanied by an enormous metabolic switch. In a resting state, the immune system maintains the existence of a diverse population of cells. Once danger is detected, specific populations need to shift to a highly activated state that runs specific transcriptional and translational programs, within a time frame of hours (reviewed in [49, 51]). In order to respond to these increased energy demands, T cells must actively acquire metabolites from their environment. For example, ligation of T cell receptor initiates cellular proliferation, whereas triggering of co-stimulatory molecules enables the uptake and usage of metabolites. Circulating growth factors, like cytokines and hormones, contribute to the ability of effector cells to switch between resting and activated states. Since the immune system comprises a multitude of different cell types and effector functions, it is of no surprise that in this tightly regulated process, specific effector functions are supported by specific metabolic pathways [51].

In a preclinical study, Nair-Gill et al. [105] investigated the immune cell specificity of PET probes for two different metabolic pathways: ^18^F-FDG for glycolysis and ^18^F-labeled 2-fluoro-d-(arabinofuranosyl)cytosine (^18^F-FAC) for deoxycytidine salvage; in response to a retrovirus-induced sarcoma. They demonstrated that the two probes had distinct patterns of accumulation: ^18^F-FDG accumulated to the highest levels in innate immune cells, while ^18^F-FAC accumulated predominantly in CD8^+^ T cells in a manner that correlated with cellular proliferation. Thus, innate and adaptive cell types differ in glycolytic and deoxycytidine salvage demands during an immune response, and this can be targeted with specific PET probes. In a similar fashion, Shu et al. [126] develop PET probes with improved metabolic stability and specificity for rate-limiting enzyme in the deoxyribonucleoside salvage pathway: deoxycytidine kinase (dCK). Given the increased body of knowledge on the metabolic fates of different cell populations, it is just a matter of time before this will be translated to monitor immune responses in clinical trials.

#### Targeting on-site immune responses in tumor tissue

The transfer of technology from tumor and preclinical imaging holds great promise. One example of such technology transfer is in the detection of apoptosis in vivo. This might also be considered in non-immunotherapeutic regimen, since it has now been demonstrated that tumor regression at least partly results from chemotherapy-induced programmed cell death and the subsequent influx and activation of immune cells [163]. The induction of tumor cell apoptosis by infiltrating immune cells precedes detectable tumor volume shrinkage (reviewed in [117]). Effective treatment should result in cell lysis, loss of membrane integrity, and increased extracellular space. These physical changes can be detected and measured using MRI. MRI is already available in the clinic and could be applied to monitoring immune responses in vivo. Among others, Annexin-V, hydrophobic cations, and caspase inhibitors have been tested as potential probes for imaging apoptosis in preclinical models. ^99m^Tc-labeled Annexin-V has been tested in clinical trials [70]. Indeed, the authors found that increased Annexin-V uptake in the tumor site early after the start of platinum-based chemotherapy in non-small cell lung cancer (NSCLC) was associated with improved clinical response. Another apoptosis marker that has been tested in humans is ^18^F-labeled 2-(5-fluoropentyhl)-2-methyl malonic acid (^18^F-ML10) [58, 64].

It is interesting to note that just 10 years ago, the field of in vivo imaging of the immune system was virtually non-existent. Five years ago, a review article covering imaging for cell tracking focused on the same techniques that we are still developing-SPIO labels for MRI, scintigraphy, and PET visualizing TK activity [161]. More recently, we have seen the preliminary introduction of these techniques in humans, the introduction of quantitative in vivo ^19^F MRI, and the distinct possibility of imminent TK-based PET in humans. We have also learned that all these probes and imaging modalities are likely to perturb the cells we are imaging. Thus, although in vivo imaging is a new field, we are already beginning to see its applications in imaging the immune system. Together with improvements in labels and imaging hardware, and the advent of multimodal imaging scanners, the future of in vivo imaging of the immune system looks bright.

## Abbreviations

^111^In: ^111^Indium
^11^C: ^11^Carbon
^124^I: ^124^Iodine
^18^F: ^18^Fluorine (positron emitter)
^19^F: ^19^Fluorine
^86^Zr: ^86^Zirconium
^99m^Tc: ^99m^Technetium
ACT: Adoptive cell transfer
ADCC: Antibody-dependent cellular cytotoxicity
APC: Antigen-presenting cell
CAR: Chimeric activation receptor
CCR7: Chemokine receptor 7
CD4: Cluster of differentiation 4
CD8: Cluster of differentiation 8
CDC: Complement-dependent cytotoxicity
CEST: Chemical exchange saturation transfer
CFA: Complete Freund’s adjuvant
CT: Computed tomography
CTL: Cytotoxic T lymphocyte
CTLA4: Cytotoxic T lymphocyte-associated protein 4
CXCL12: C-X-C motif ligand 12 (aka SDF-1)
CXCR4: C-X-C chemokine receptor 4 (aka CD184)
DC: Dendritic cell(s)
DCE-MRI: Dynamic contrast-enhanced magnetic resonance imaging
dCK: Deoxycytidine kinase
DW-MRI: Diffusion weighted magnetic resonance imaging
EC: Endothelial cells
FAC: 2-fluoro-d-(arabinofuranosyl)cytosine
FDA: Food and Drug Association
FDG: Fluoro-2-deoxy-2-D3′-fluoro-3′-deoxy-thymidine-glucose
FLT: 3′-fluoro-3′-deoxy-thymidine
Gd: Gadolinium
GFP: Green fluorescent protein
HPV: Human papilloma virus
HU: Hounsfield Unit
i.d.: Intradermal
i.l.: Intralymphatic
i.n.: Intranodal
i.v.: Intravenous
IFNα: Interferon-alpha
IFNγ: Interferon-gamma
IL-2: Interleukin-2
LN: Lymph node
LYVE-1: Lymphatic vessel endothelial hyaluronan receptor-1
mAb: Monoclonal antibody
MHC: Major histocompatibility complex
ML10: 2-(5-fluoropentyhl)-2-methyl malonic acid
MRI: Magnetic resonance imaging
NK: Natural killer
NSCLC: Non-small cell lung cancer
PAMPs: Pathogen-associated molecular pattern
PD1: Programmed death receptor 1
PET: Positron emission tomography
PRG: PET-reporter gene
PRP: PET-reporter probe
RECIST: Response evaluation criteria in solid tumors
RGD: Arginyl-glycyl-aspartic acid
s.c.: Subcutaneous
SPECT: Single-photon emission computed tomography
SPIO: Superparamagnetic iron oxide
TAA: Tumor-associated antigen
TCR: T cell receptor
Th1/2: T helper 1/2
TIL: Tumor infiltrating lymphocyte
TK-1/2: Thymidine kinase 1/2
TNF: Tumor necrosis factor
Treg: T regulatory
VEGF: Vascular endothelial growth factor
